# Avapritinib in advanced gastrointestinal stromal tumor: case series and review of the literature from a tertiary care center in India

**DOI:** 10.2144/fsoa-2020-0178

**Published:** 2021-01-19

**Authors:** Saurav Verma, Rohit Reddy, Sheragaru Hanumanthappa Chandrashekhara, Shamim Ahmed Shamim, Sarthak Tripathy, Sameer Rastogi

**Affiliations:** 1 Department of Medical Oncology, Institute Rotary Cancer Hospital, All India Institute of Medical Sciences, New Delhi 110029, India; 2 Department of Radiodiagnosis, Institute Rotary Cancer Hospital, All India Institute of Medical Sciences, New Delhi 110029, India; 3 Department of Nuclear Medicine, All India Institute of Medical Sciences, New Delhi 110029, India; 4 Sarcoma Medical Oncology Clinic, Department of Medical Oncology, Institute Rotary Cancer Hospital, All India Institute of Medical Sciences, New Delhi 110029, India

**Keywords:** avapritinib, gastrointestinal stromal tumors, GIST, *PDGFRA* D842V

## Abstract

The therapeutic landscape in advanced gastrointestinal stromal tumor has evolved. Avapritinib and ripretinib have now been approved by the US FDA for platelet-derived growth factor alpha D842V-mutant and refractory gastrointestinal stromal tumor patients, respectively. Here we report five patients who have been on avapritinib under an expanded access program. Response assessment was available for four patients – a partial response in two patients and stable disease in one, while one patient had progressive disease. Though preliminary results of the VOYAGER trial have shown less activity of avapritinib and no significant difference in progression-free survival when compared with regorafenib, avapritinib may show some clinical benefit in a subset of patients refractory to approved therapies. We share our experience of five cases, with clinical benefit in three. We believe avapritinib should be further evaluated in clinical trials.

## Lay abstract

The new therapeutic options, avapritinib and ripretinib, alongside the molecular landscape of driver mutations in gastrointestinal stromal tumor present an opportunity for personalized treatment. We report five cases of advanced and refractory gastrointestinal stromal tumor who received avapritinib under an expanded access program. Two patients had a partial response; one each had a stable and progressive disease. Response was not available for one patient. In the VOYAGER trial, avapritinib failed the primary end point but showed clinical activity and hence avapritinib merits further evaluation. Our experience with avapritinib supports further testing of this drug.

Gastrointestinal stromal tumors (GISTs) are neoplasms of mesenchymal origin that occur most commonly in the stomach and small intestine. Most (90%) GISTs harbor either *c-KIT* (~75%) or platelet-derived growth factor alpha (*PDGFRA*) (10–20%) mutations. Approval of imatinib in advanced GISTs led to a dramatic improvement in outcomes with the median progression-free survival (PFS) being around 2 years and a median overall survival (OS) around 5 years, with a fraction of patients doing well for as long as 10 years [1]. However, the sensitivity of the disease to imatinib is a function of the mutational status of the tumor with patients harboring *c-KIT* exon 11 mutation showing better outcomes as compared with those with c-KIT exon 9 mutation. Similarly, wild-type and *PDGFRA* D842V mutated GISTs are relatively insensitive to imatinib [2]. For patients having progression on imatinib, the subsequent second- and third-line US FDA-approved drugs are sunitinib and regorafenib respectively [3,4].

Avapritinib is a selective tyrosine kinase inhibitor targeting *PDGFRA* including *PDGFRA* D842V mutations along with *c-KIT* mutations in exon 11 and exon 17. It also has good activity in activating loop mutations. In January 2020, the FDA approved avapritinib with Breakthrough Therapy designation for patients with GISTs harboring *PDGFRA* exon 18 mutations including D842V following the NAVIGATOR trial, a Phase I multicenter single-arm trial [5]. The primary end point in this trial was safety. For patients harboring the *PDGFRA* D842V mutation (n = 56), the overall response rate (ORR) was 88%. The median duration of response was not yet reached with a median follow-up of 15.9 months [6].

Ripretinib is a pan-KIT and *PDGFRA* switch-control inhibitor and has activity at the ATP-binding pocket as well as the activation loop. In the recently published INVICTUS trial, 129 patients with advanced GIST were randomized to ripretinib or placebo. The ripretinib arm had a median PFS of 6.3 months compared with 1 month in the placebo arm and it significantly reduced the risk of disease progression or death by 85% (hazard ratio of 0.15, p < 0.0001). Median OS was also superior in the ripretinib arm as compared with the placebo, 15.1 months versus 6.6 months. Based on this trial, ripretinib was approved by the FDA as a fourth-line therapy in advanced GIST [7].

So far, the above-mentioned developments have only touched the developed world. We are reporting our experience with five patients who were given avapritinib through a compassionate use program.

## Materials & methods

This is a retrospective study evaluating patients with metastatic GIST who progressed post multiple lines of treatment and were treated with avapritinib 300 mg per day starting from September 2019 and followed till August 2020. Avapritinib was accessed through a support program on a compassionate basis. The pathology of all the cases was reviewed by a sarcoma pathologist.

The dose of avapritinib used in the clinic was 300 mg per day (as per the trials on avapritinib). Data was collected through hospital records (including the age, sex, site, mutation status, prior lines of treatment, metastatic lesions, responses and outcomes) and analyzed. The statistical analysis was done through SPSS 23 (SPSS, IL, USA). Nominal data are provided as number (%) and continuous data as median (range). PFS was calculated from the date of start of treatment to the first date of documented progressive disease (PD) or the date of death from any cause.

## Results

### Case 1

**Figure 1.**
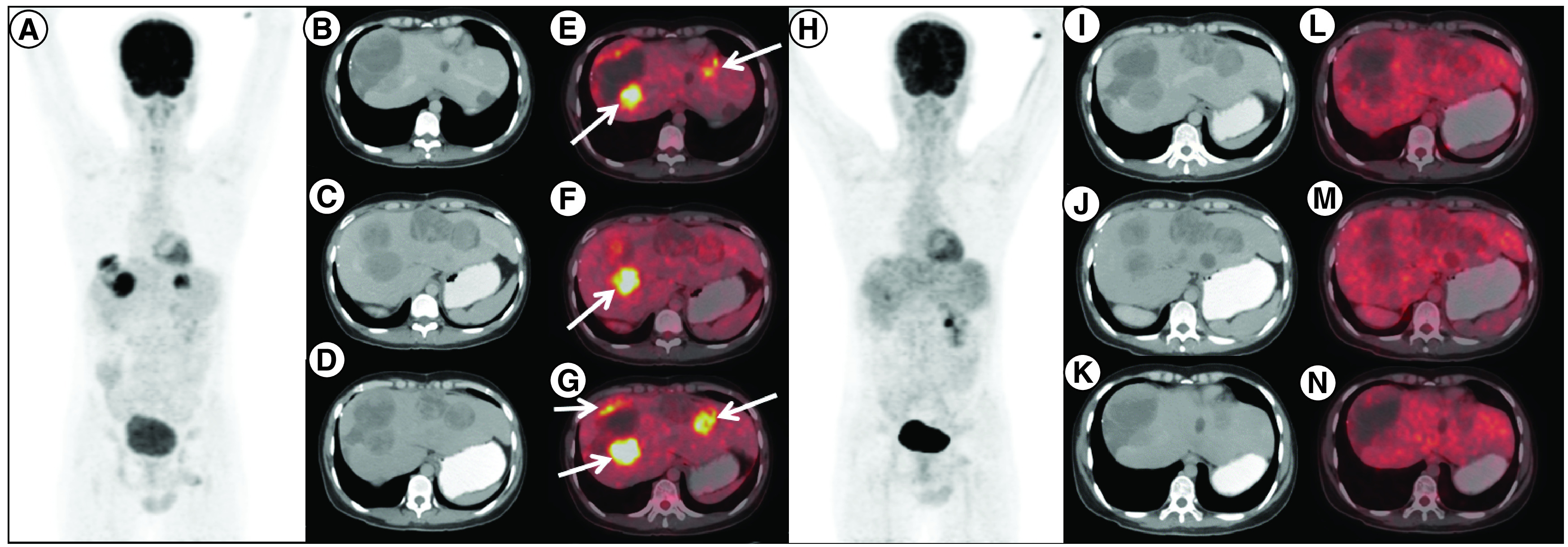
Response to imatinib, sunitinib and regorafenib therapy in case 1. ^18^F-Fluorodeoxyglucose (FDG) positron emission tomography/computed tomography (PET/CT) done post imatinib, sunitinib and regorafenib therapy in patient A. **(A)** maximum intensity projection, **(B, C & D)** axial contrast enhanced CT and **(E, F & G)** fused PET/CT images showing multiple solid cystic masses with increased FDG uptake in the enhancing solid component (arrows) suggestive of metabolically active liver metastases. Follow-up ^18^F-FDG PET/CT done 3 months after initiation of avapritinib therapy, which revealed resolution of metabolic activity in the corresponding lesions **(H–N)** with hypodense/cystic changes in the previously enhancing lesions, suggestive of complete metabolic response.

**Figure 2.**
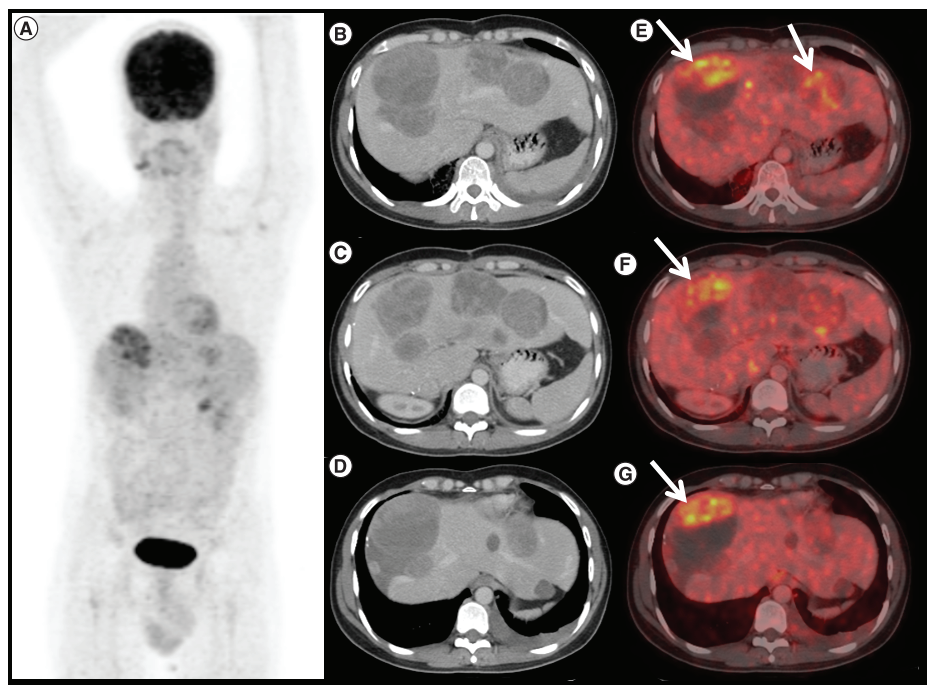
Response to avapritinib therapy in case 1. ^1^^8^F-FDG positron emission tomography/computed tomography (PET/CT) done 6 months after avapritinib therapy in patient A. **(A)** Maximum intensity projection, **(B, C & D)** axial contrast enhanced CT and **(E, F & G)** fused PET/CT images shows increase in size of the liver lesions with FDG avid enhancing areas in the periphery of the lesion (arrows), suggestive of progressive disease.

A 28-year-old male presented with jejunal GIST with liver metastases (mutation testing not available at baseline). He underwent en-bloc resection of primary tumor with a wide excision of liver metastases and was started on imatinib. Post 5 years of therapy, he had disease progression (increase in liver metastases and new peritoneal metastases). Mutation testing was suggestive of exon 17 mutation in the *c-KIT* domain. He was started on sunitinib 37.5 mg daily and after 2 months there was a radiological progression following which he was started on regorafenib. Regorafenib provided disease control for almost 6 months. At this point, imatinib was reintroduced but there was no symptomatic benefit. After a discussion in the tumor board, the patient was started on avapritinib. He tolerated avapritinib well with greying of hairs and transaminitis (grade 2). A scan was done post 3 months for response evaluation and showed a complete metabolic response and stable disease (Figure 1). The therapy was continued, however, another response assessment was done at 6 months showed PD (Figure 2).

### Case 2

**Figure 3.**
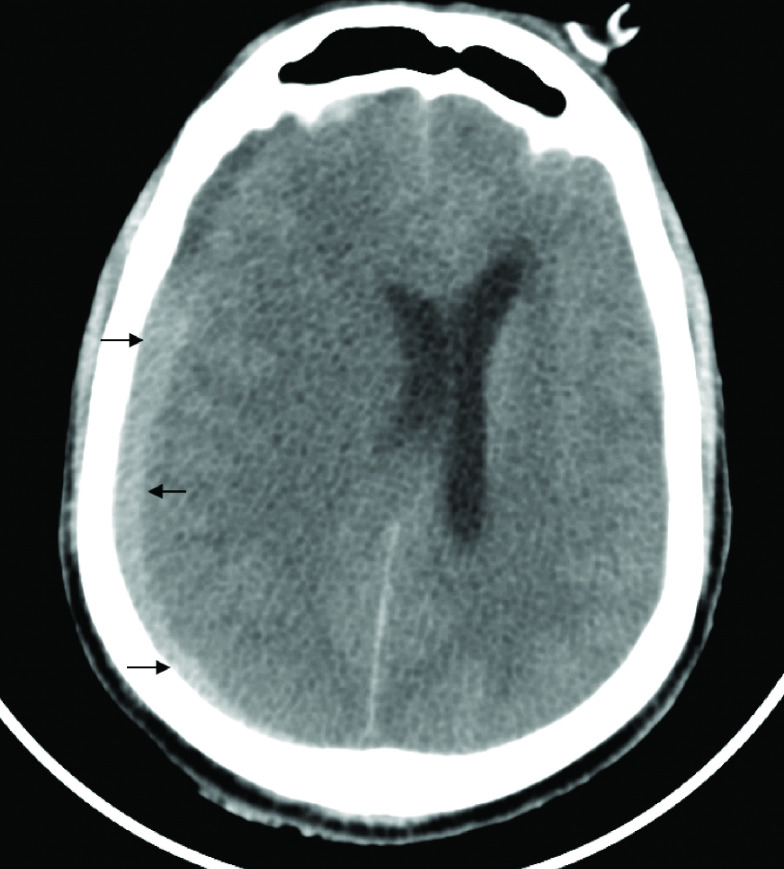
Subdural haemorrhage in case 2. Computed tomography head of patient B showing biconcave hyperdensity in the right parieto-occipital location with associated midline shift suggestive of subdural hemorrhage.

A 49-year-old male with duodenal GIST and liver metastases (*c-KIT* exon 11 mutation positive) was started on imatinib at a dose of 400 mg once a day. After 2 years of therapy, he had PD with an increase in liver lesions and was subsequently started on sunitinib. He underwent surgical resection of primary tumor along with metastatic liver lesions at the same time and, sunitinib was continued. Progression was noted at 6 months and regorafenib was started. After 3 months the liver lesions increased and he was initiated on avapritinib. He had a partial response at 2 months of therapy with avapritinib and stable disease at 5 months. However, he had a progression at 8 months and a grade 4 subdural hemorrhage (Figure 3).

### Case 3

A 40-year-old male with gastric GIST and liver metastases (*c-KIT* exon 9 mutation positive) was started on imatinib 400 mg once a day. After a disease control for 18 months, liver lesions progressed. He was started on sunitinib 37.5 mg daily which provided control for another 5 months. He was subsequently started on regorafenib 80/120 mg alternate day (as there was a concern for toxicity). He again had a PD after 4 months on regorafenib. After a discussion in the tumor board, avapritinib was started. He received avapritinib for 1 month, with a treatment interruption of 3 months due to an ongoing corona pandemic. He was again restarted on the avapritinib, however response assessment at 1 month showed PD.

### Case 4

A 60-year-old lady presented with jejunal GIST in April 2017 (mutation status not known). The tumor was infiltrating the adjacent mesentery, hepatic flexure and abutting the SMV. She was started on imatinib 400 mg per day and had a partial response. She underwent surgery in January 2018 but was deemed unresectable post-laparotomy and continued on imatinib. In January 2019 her disease progressed. She was treated with sunitinib and regorafenib and had progression on both therapies. She was started on avapritinib but died after 1 month due to disease progression.

### Case 5

**Figure 4.**
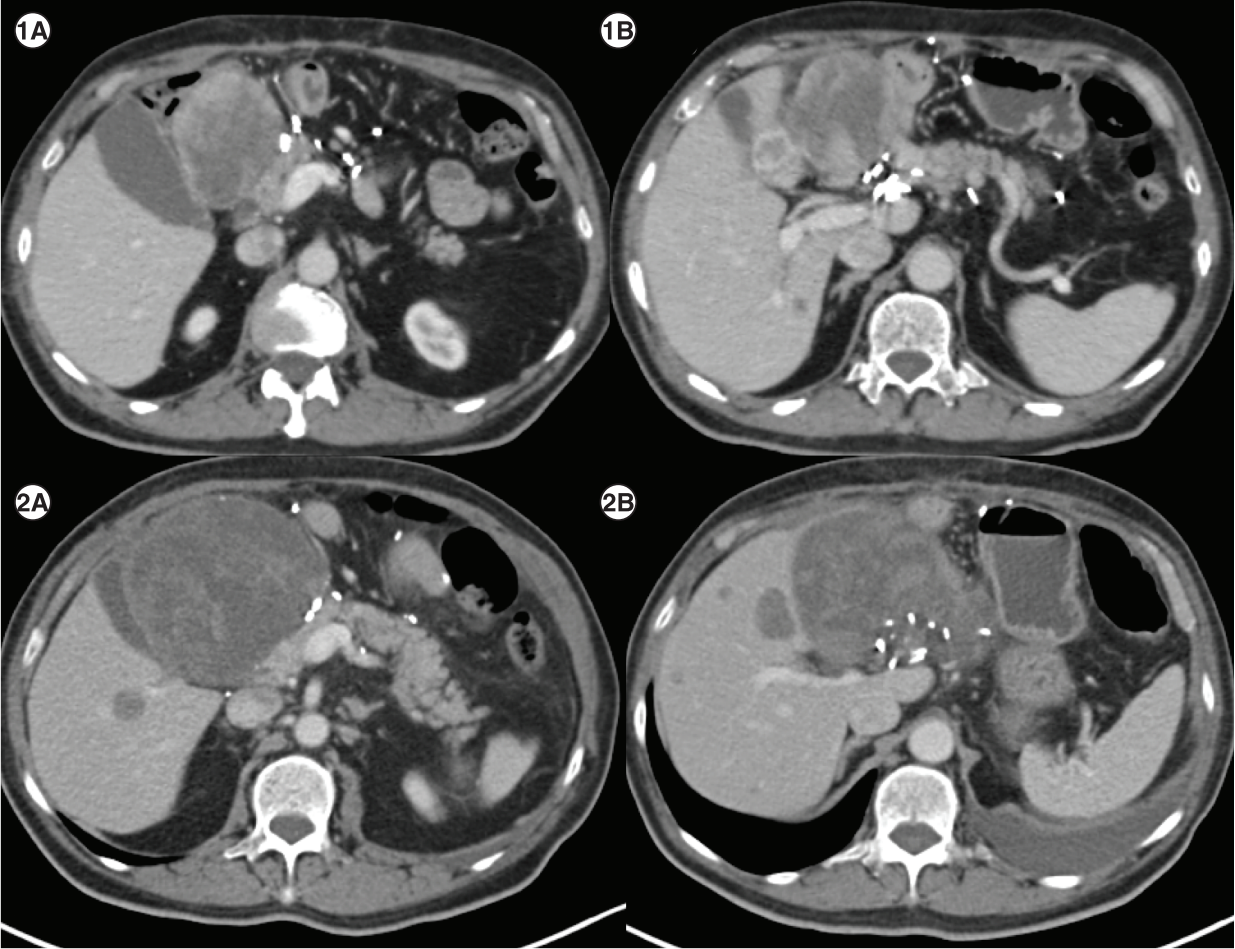
Response to imatinib in case 5. **(1A)** Axial computed tomography abdomen of patient E showing a heterogenous mass measuring 7.1 × 6.6 cm in the surgical bed in the gastro-hepatic region and a hypodense lesion in segment IVB of liver **(1B)** measuring1.5 × 1.8 cm. **(2A)** Axial CT abdomen of first follow-up scan showing a heterogenous mass measuring10.2 × 10.7 cm in the surgical bed in the gastro-hepatic region and a hypodense lesion in segment IVB of the liver measuring 2.9 × 2.8cm **(2B)**, suggestive of disease progression.

**Figure 5.**
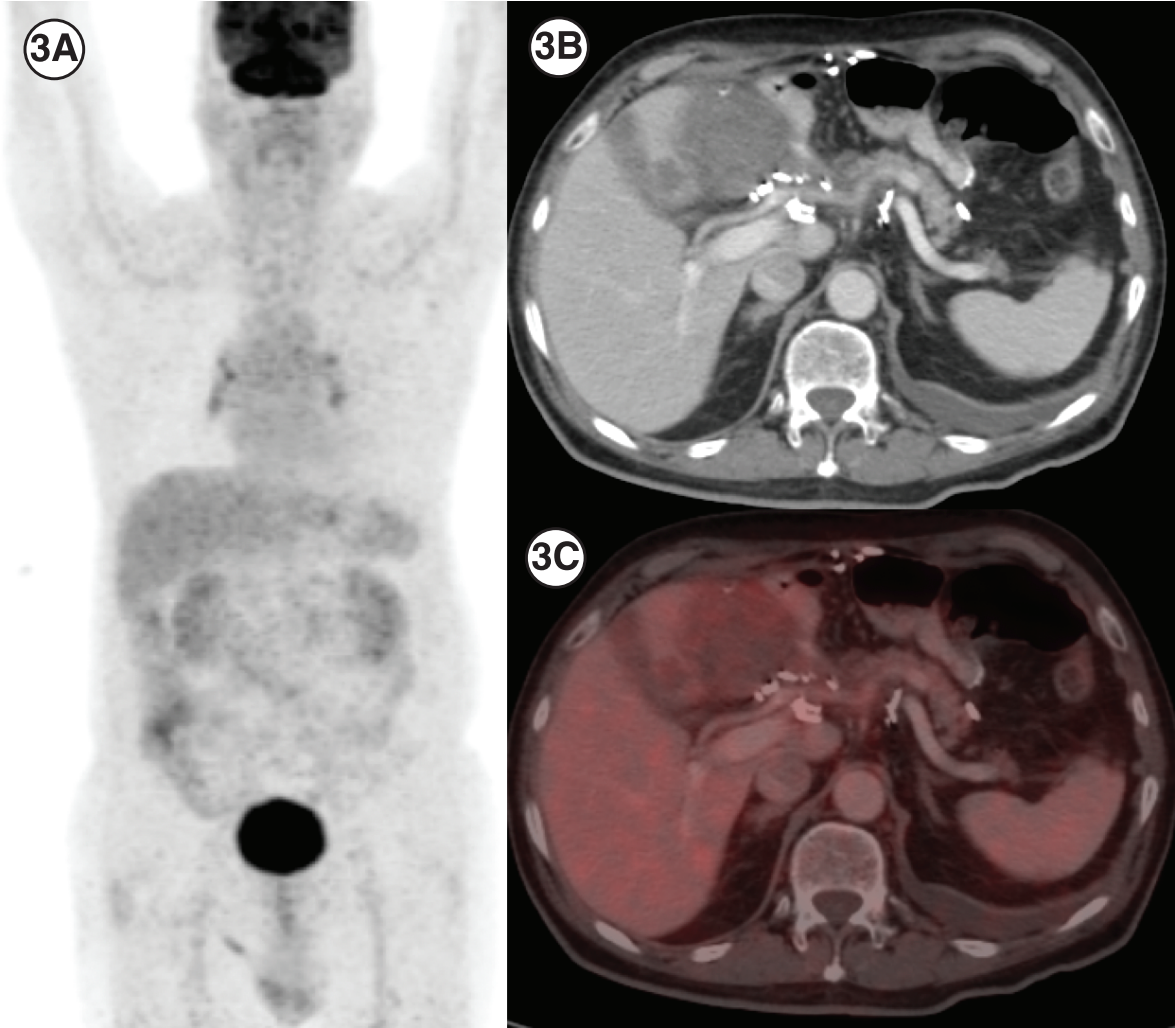
Response to avapritinib in case 5. **(3A)** In a patient with *PDGFRA* D842V mutation (patient E), maximum intensity projection image of ^18^F-Fluorodeoxyglucose (FDG) positron emission tomography/computed tomography (PET/CT) showing physiologic biodistribution in the brain, liver, kidneys and urinary bladder. **(3B)** Axial CT abdomen showing a heterogenous mass measuring 5.5 × 6.0 cm in the surgical bed in the gastro-hepatic region and a hypodense lesion in the segment IVB of the liver 2.1 × 2.0 cm with no significant FDG uptake in any of the lesions on fused PET-CT images, suggestive of partial response **(3C)**.

A 76-year-old gentleman presented with gastric GIST in November 2018. He was started on neoadjuvant imatinib because of locally advanced disease at presentation. However, he progressed (Figure 4) and underwent subtotal gastrectomy with gastrojejunostomy. Postsurgery he was kept on observation. In March 2020 he had a recurrence with a large lesion in the operative bed which was abutting pancreatic head, bowel loops and inferior liver surface; multiple large gastro-hepatic nodal deposits and liver metastases. Mutation testing was suggestive of *PDGFRA* D842V mutation. He was started on avapritinib and response assessment at 3 months was suggestive of a partial response (Figure 5).

Baseline characteristics of the five patients have been depicted in Table 1. The median age of our patients was 49 years (range: 28–76). The sites of the primary tumor were stomach and duodenum in two patients each, with one patient having a jejunal primary. The mutation analysis of our cohort is depicted in Table 1. All the patients were extensively treated with median lines of treatment of three (range: 2–4). Among them, four (80%) patients showed a partial response with the first line of treatment (imatinib). One patient (20%), who had *PDGFR* D842V mutation, had PD on imatinib. All patients (n = 5) progressed on sunitinib with one patient (20%) experiencing grade 3 toxicities who could not tolerate the drug, requiring discontinuation. Two (40%) of five patients on regorafenib had a partial response. The most common site of metastasis was the liver (n = 5; 100%); next being peritoneal and serosal deposits (n = 4; 80%). The average sites of metastasis were three (range: 2–4) indicating a heavy burden of disease.

**Table 1.** Base line characteristics of patients.

	Primary site of tumor	Age (years)/sex	Mutation	Response to imatinib	Response to sunitinib	Response to regorafenib	Other treatments tried	Sites of metastasis
A	Jejunum	28/male	*c-KIT* exon 17	PR	PD	PD	Progressed on immunotherapy	Liver, omental, serosa
B	Duodenum	49/male	*c-KIT* exon 11	PR	PD	PD	Progressed on immunotherapy	Liver, omental, serosa, bone, nodes
C	Stomach	40/male	*c-KIT* exon 9	PR	PD	PR		Liver, omental, bone
D	Duodenum	60/female	Not known	PR	PD	PD	Progressed on pazopanib	Liver, omental, serosa
E	Stomach	76/male	*PDGFRA* D842V	PD	Could not tolerate	PR		Liver, serosa

PD: Progressive disease; PDGFRA: Platelet-derived growth factor alpha; PR: Partial response; SD: Stable disease.

**Figure 6.**
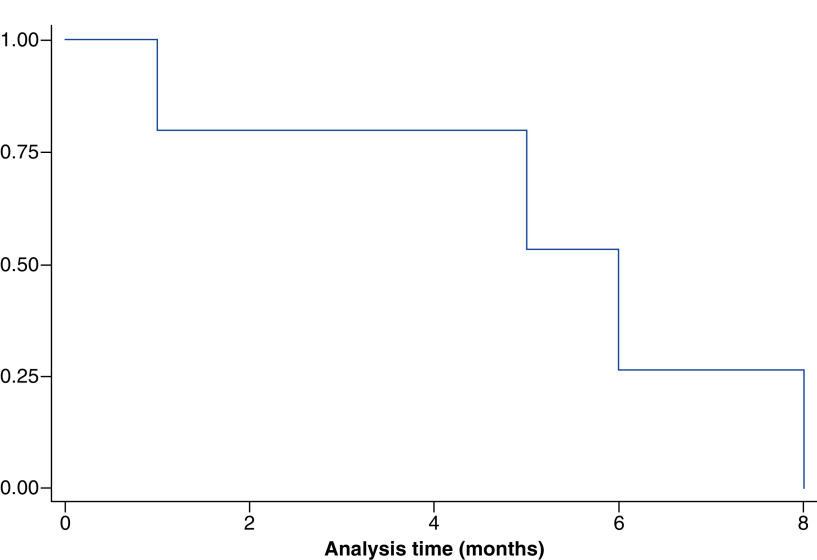
Progression-free survival.

Table 2 depicts the avapritinib characteristics of the five patients. The best response to avapritinib was stable disease in one patient (20%: patient A), a partial response in two patients (40%: patient B and E), while one patient had a progression. Response assessment was not available for one patient. At the time of analysis, three patients have progressed on avapritinib and one had died. The median PFS on avapritinib was 6 months (range; 1–8) (Figure 6). The drug was mostly tolerable in our patient cohort with all of them (n = 5) experiencing grade 2 toxicities, one (20%) patient experiencing grade 3 toxicities and one had a grade 4 subdural hemorrhage. The most common toxicity observed was skin toxicity in the form of maculopapular rash (n = 3; 60%), but none of them required dose interruptions. Grade 2 hyperbilirubinemia was seen in two (40%) patients and one patient (20%) had a moderate pleural effusion.

**Table 2.** Avapritinib characteristics including response rates, progression free time, outcomes and toxicities.

	Dose	Response	Progression-free time	Outcomes	Toxicities
A	300 mg	Initial SD at 3 months followed by PD at 6 months	6 months	On ripretinib	Grade 3 rash, greying of hair, grade 2 hyperbilirubinemia, grade 2 anemia
B	300 mg	Initial PR at 2 months, SD at 5 months followed by PD at 8 months	8 months	Planned for ripretinib	Grade 2 rash, periorbital puffiness, grade 4 SDH
C	300 mg	PD	5 months	Planned for ripretinib	Grade 2 rash
D	300 mg	Not available	1 months	Death	Grade 2 hyperbilirubinemia
E	300 mg	PR	3 months	On avapritinib	Periorbital puffiness, pleural effusion

PD: Progressive disease; PR: Partial response; SD: Stable disease; SDH: Subdural hemorrhage.

## Discussion

Approval of avapritinib has advanced precision oncology in GIST and reiterates the importance of mutation testing in all patients. The younger age at presentation in our patients is similar to other studies of GIST from India. All patients (n = 5) had previously received imatinib, sunitinib and regorafenib. Among them, four (80%) patients had a partial response to imatinib. The patient with *PDGFRA* D842V mutation had progressed on imatinib and showed a partial response to avapritinib. In a study by Yoo *et al.* *PDGFRA* D842V mutated patients had significantly worse PFS as compared with *PDGFRA* D842V wild-type patients (3.8 vs 29.5 months; p < 0.001), highlighting the imatinib refractory nature of this subtype [8].

Our study adds further to the evidence for the use of avapritinib in patients with GIST with non-*PDGFR* D842V mutation. Of all patients with non-*PDGFRA* D842V tumors (n = 4), two (50%) patients received immunotherapy with single-agent nivolumab. In a Phase II study by Singh *et al.* evaluating nivolumab in one arm and nivolumab with ipilimumab in another arm, there was a modest benefit with the use of immunotherapy showing a median PFS of around 8.5–9 weeks in both arms [9]. However complete results, including the utility of biomarkers, are not yet fully reported. The VOYAGER trial, an international, multicenter, open-label, randomized study explored avapritinib in a third or fourth line setting in comparison to regorafenib [10]. A total of 476 patients were randomized (240 patients in the avapritinib arm and 236 in the regorafenib comparator arm) with the primary end point as PFS. There was no significant difference in median PFS between the avapritinib arm (4.2 months) and the regorafenib arm (5.6 months). The overall response in the avapritinib arm was 17% as compared with 7% response in the regorafenib arm [11]. A good response rate and reasonable PFS of 4.2 months show that this drug is active in the subset of unresectable/metastatic third and fourth-line GIST [11]. Hence, this drug should be further tested in this subset [12].

The NAVIGATOR trial evaluated avapritinib in advanced GIST including patients with KIT and PDGFRA D842V mutation. The ORR in the 4L+ population was 22% with a median duration of response of 10.2 months. It showed unrivaled responses in D842V and other exon 18 mutant (non-D842V) *PDGFRA* GIST with ORR being 88 and 84%, respectively [6].

The dose of 300 mg per day was well tolerated in our five patients except one (20%) patient with a grade 3 rash and one with a grade 4 subdural hemorrhage. Rash (n = 3; 60%) and hyperbilirubinemia (n = 2; 40%) were the most common grade 1 or 2 toxicities observed. Two patients (n = 2; 40%) had periorbital edema. In the Phase I NAVIGATOR trial, the adverse effects were most commonly grade 1–2 at 300 mg dose, most common adverse effects being nausea (69%), diarrhea (41%), decreased appetite (38%) and fatigue (38%). Sixteen percent of patients had periorbital edema and 6% had hyperbilirubinemia. Rash was seen in 3% of patients at 300 mg but in 33% at 600 mg dose [6]. Two adverse events of special interest were observed in the NAVIGATOR trial – cognitive effects, and intracranial bleeding. In NAVIGATOR trial, two (2%) of 82 patients had grade 3 intracranial bleeding events (one cerebral hemorrhage and one intracranial hemorrhage), which led to treatment discontinuation. Similar events of intracranial hemorrhage were noted in preclinical studies with avapritinib. None of our patients had any noted cognitive effects. One patient (case 4) had a grade 4 subdural hemorrhage. He did not have any history of trauma and was not on anticoagulation. Hence, we believe it to be possibly related to avapritinib-related platelet dysfunction. There have been rare reports of subdural hemorrhage in GIST with imatinib, and whether KIT and PDGFRA inhibition is implicated in such cases is unknown [13,14].

A study showed that avapritinib inhibited tumor progression significantly better than regorafenib in imatinib-resistant cell lines (exon 11/17 line) [15]. Patient A and patient B, with *c-KIT* exon 17 and exon 11 mutation respectively, showed benefit with avapritinib. Patient C with *c-KIT* exon 9 mutation had an interruption in treatment and had disease progression.

One patient was lost to follow-up because of difficulty in traveling and poor access to healthcare facilities amidst lockdown due to coronavirus disease 2019 (COVID-19), casting light on the problems faced by patients during a pandemic. The lack of mutational testing in most cities further limits our options of identifying patients who can benefit from personalized medicine.

The novel *KIT* and *PDGFRA* inhibitor ripretinib improved PFS in heavily treated patients in the INVICTUS trial; becoming a lifeline in fourth line treatment of GIST albeit an OS benefit needs to be demonstrated [7]. To harness its benefit in second line post-progression on imatinib, it is being pitched against sunitinib in INTRIGUE study (NCT03673501).

Apart from avapritinib, there are other few promising drugs in the *PDGFRA* D842V mutated subset of patients. Heinrich *et al.* demonstrated the activity of crenolanib in GIST cell lines containing the D842V mutation *in vitro* [16]. A Phase III clinical trial evaluating crenolanib in this population is ongoing (NCT02847429) [17]. Olaratumab was evaluated in a phase 2 trial in previously treated patients with metastatic GISTs and showed a meaningful PFS of 32 weeks in the *PDGFRA* D842V mutated cohort [18].

Other than *PDGFRA* D842V mutation other personalized therapies are also being explored in GIST. Wild-type GISTs – a majority of which are subdural hemorrhage deficient and have global DNA hypermethylation is sensitive to FGFR inhibition; validating the role of epigenetic factors in oncogenesis in GISTs and paving the way to the development of combined FGFR and KIT inhibition [19]. A prospective Phase II trial of temozolomide subdural hemorrhage-mutant/deficient advanced/metastatic GIST is currently enrolling patients (NCT03556384) [20].

## Conclusion

The era of personalized medicine in recurrent and metastatic GISTs has progressed with the elucidation of new pathways of carcinogenesis and resistance; leading to the development of new drugs. We believe that the trials with these novel drugs should be open to developing countries, where the majority of patients do not have access to these new drugs.

## Future perspective

GIST is a heterogenous disease driven by specific molecular drivers and as such personalized treatment will be the way forward. Avapritinib has heralded hope as a first-line treatment for *PDGFRA* D842V mutated GIST patients, and shows activity in the third and fourth lines of treatment. Avapritinib should be tested in earlier lines of therapy with preplanned molecularly defined subgroups. Promising drugs like crenolanib and olaratumab are being tested in trials and the future looks promising.

**Executive summary**

- Gastrointestinal stromal tumor (GIST) is a rare mesenchymal tumor that arises mainly in the stomach and small bowel.
- GIST is a heterogenous disease with specific driver mutations. Around 90% of GISTs harbor driver mutations in KIT and platelet-derived growth factor alpha (PDGFRA).
- Recently, the US FDA approved avapritinib (for *PDGFRA* D842V-mutant GIST) and ripretinib (for refractory GIST). These drugs have added to the previously existing treatment options – imatinib, sunitinib and regorafenib.
- We report five cases of advanced/refractory GIST who received avapritinib under an expanded access program.
- Three patients had a *c-KIT* and one *PDGFRA* D842V mutation.
- The best response to avapritinib was a partial response in two patients, stable disease in one and progressive disease in one. Response was not available for one patient. The median progression-free survival was 6 months.
- Avapritinib was well tolerated except grade 4 subdural hemorrhage in one patient which was possibly related to avapritinib.
- The VOYAGER trial could not demonstrate a progression-free survival benefit with avapritinib compared with regorafenib, however, avapritinib showed clinical activity in these patients.
- The approval of avapritinib was on the basis of Phase I NAVIGATOR trial which evaluated avapritinib in advanced GIST, including patients with KIT and PDGFRA D842V mutation.
- Two specific adverse events were noted in NAVIGATOR trial – cognitive impairment and intracranial hemorrhage.
- Avapritinib also shows activity in imatinib-resistant cell lines (exon 11/17 line).
- Avapritinib is a promising drug and its clinical benefit in second and third line treatment of advanced GIST should be explored in future trials with enrichment of trial population by specific molecular subgroups.
- The future of GIST lies in personalized therapy and looks promising, with progress in understanding of molecular drivers and secondary resistance mechanisms which can be targeted by novel drugs.
